# Assessment by Matrix‐Assisted Laser Desorption/Ionization Time‐of‐Flight Mass Spectrometry of the Effects of Preanalytical Variables on Serum Peptidome Profiles Following Long‐Term Sample Storage

**DOI:** 10.1002/prca.201700047

**Published:** 2018-03-02

**Authors:** Sachio Tsuchida, Mamoru Satoh, Hiroshi Umemura, Kazuyuki Sogawa, Masaki Takiwaki, Takayuki Ishige, Yui Miyabayashi, Yuuya Iwasawa, Sohei Kobayashi, Minako Beppu, Motoi Nishimura, Yoshio Kodera, Kazuyuki Matsushita, Fumio Nomura

**Affiliations:** ^1^ Division of Clinical Mass Spectrometry Chiba University Hospital Chiba Japan; ^2^ Departments of Dermatology Okayama University Graduate School of Medicine, Dentistry and Pharmaceutical Sciences Okayama Japan; ^3^ Department of Biochemistry School of Life and Environmental Science Azabu University Sagamihara Japan; ^4^ Department of Molecular Diagnosis Graduate School of Medicine Chiba University Chiba Japan; ^5^ Laboratory of Biomolecular Dynamics Department of physics School of Science Kitasato University Chiba Japan

**Keywords:** ClinProtRobot, long‐term sample storage, MALDI‐TOF MS, serum peptidome

## Abstract

**Purpose:**

Human serum and plasma are often used as clinical specimens in proteomics analyses, and peptidome profiling of human serum is a promising tool for identifying novel disease‐associated biomarkers. Matrix‐assisted laser desorption/ionization time‐of‐flight mass spectrometry (MALDI‐TOF MS) is widely used for peptidomic biomarker discovery. Careful sample collection and handling are required as either can have a profound impact on serum peptidome patterns, yet the effects of preanalytical variables on serum peptidome profiles have not been completely elucidated. The present study investigated the effects of preanalytical variables, including storage temperature, duration (up to 12 months), and thawing methods, on MALDI‐TOF MS‐based serum peptidome patterns.

**Experimental design:**

Aliquots of serum samples were pretreated with weak cation exchanger magnetic beads using an automated ClinProtRobot system and then analyzed by MALDI‐TOF MS.

**Results:**

A number of significant differences in peak intensities were observed depending on sample processing variables.

**Conclusions and clinical relevance:**

These peaks can be used as sample quality markers to assess the effects of long‐term storage on serum peptidome profiles using MALDI‐TOF MS.

## 1 Introduction

Protein and peptide biomarker discovery is a major interest in proteome research, and profiling methods based on matrix‐assisted laser desorption/ionization time‐of‐flight (MALDI‐TOF) mass spectrometry (MS) analysis of serum samples are promising tools in this area.^1^, ^2^, ^3^ Previously, we applied automation and robotics to the ClinProt MALDI‐TOF MS system and detected novel biomarkers in serum samples for multiple sclerosis, alcohol abuse, chronic thromboembolic pulmonary hypertension, and early gastric cancer.^4^, ^5^, ^6^, ^7^

Sample collection and handling procedures can have a profound impact on the serum proteome, and we previously demonstrated that the time between venipuncture and serum preparation affected serum peptidome patterns.^8^ To minimize the possible degradation of serum proteins, serum samples should be frozen at the coldest possible temperature, particularly if they are to be stored for an extended period.^9^, ^10^, ^11^

Standardizing preanalytical serum sample procedures can prevent marked variations in serum proteome profiling. However, despite obvious discrepancies between reports evaluating protein profiling using MALDI‐TOF MS, only a few studies have focused on the effect of preanalytical conditions when profiling low‐molecular‐weight serum proteins.^12^, ^13^, ^14^

Previous studies have only investigated relatively short‐term storage (up to 3 months). Serum samples might be stored at −20 °C instead of −80 °C, yet it remains unclear how the serum proteome and peptidome patterns of serum samples stored for up to 12 months at −20 °C differ from those stored at −80 °C.

Moreover, the appropriate conditions for long‐term storage and processing of serum samples for peptidome analysis have not been fully established.

**Clinical Relevance**

This study aimed to assess the long‐term (up to 12 months) effects of sample storage under various conditions and also the ways to freeze samples on serum peptidome patterns using MALDI‐TOF MS. The results may have clinical relevance for diagnostic biomarker discovery using stored clinical specimens. Based on our results, we would recommend to use samples stored at −80 °C or in liquid nitrogen instead of those stored at −20 °C for biomarker discovery study targeting serum peptides. In terms of biobank study in which clinical specimens are going to be stored for years, it remains to be clarified which storage condition is most appropriate. When we use serum specimens that are stored frozen for peptidomic analysis, the manner in which the specimen is thawed may affect the results. The results of this study indicate that there was no difference in the relative intensity of each peak between samples thawed on ice, at room temperature, or at 37 °C when stored for 3, 6, or 12 months at −80 °C or in liquid nitrogen.

The number of long‐term biobanks has recently increased.^15^, ^16^ Biobanking is important for long‐term clinical patient follow‐up.^15^, ^16^ Biobanks store body fluids, such as blood, saliva, urine, and cerebrospinal fluid (CSF), for long periods and are essential for biological and biomedical research and for laboratory diagnostics.^14^, ^15^ Serum samples stored in biobanks are often used in multiple serological or high‐throughput proteomic comparative case–control studies.^17^, ^18^ Successful long‐term storage of a serum sample depends on quality control and assurance.^19^, ^20^

The aim of this study was to assess the long‐term (up to 12 months) effects of preanalytical variables, specifically, the effect of storage temperature and duration on serum peptidome patterns within frozen samples as assessed by MALDI‐TOF MS. Three different storage temperatures were compared: −20 °C, −80 °C, and −196 °C (liquid nitrogen). In addition, we assessed how thawing conditions alter the serum peptidome profiles of the samples. Aliquots of the variously frozen, stored, and thawed serum samples were further characterized by sodium dodecyl sulfate polyacrylamide gel electrophoresis (SDS‐PAGE) analysis, followed by densitometry estimation.

## 2 Experimental Section

### 2.1 Samples and Handling Procedures

Venous blood samples were obtained from eight apparently healthy volunteers (four males, four females) with a mean age of 34.5 ± 5.9 years. All volunteers provided informed consent before participation in this study. Whole blood samples were collected in Vacutainer tubes containing SiO_2_ as a coagulation enhancer, left at room temperature for 30 min, and then centrifuged at 1200 × *g* for 15 min at 4 °C to obtain serum. The serum samples were frozen in liquid nitrogen, then stored at −20 °C, −80 °C, or in liquid nitrogen until analysis.

### 2.2 Magnetic Bead Serum Sample Preparation for the MALDI‐TOF MS ClinProt System

We used weak cation exchange (WCX) magnetic beads (Bruker Daltonics GmbH, Bremen, Germany) and a ClinProtRobot automatic robot (Bruker Daltonics) to fractionate the serum peptidomes according to the manufacturer's protocol. Parathyroid hormone (MW 3718) and muscarinic toxin 1 (MW 7509) synthetic peptides were purchased from Peptide Institute, Inc. (Osaka, Japan). Ten microliters of parathyroid hormone peptide and muscarinic toxin 1 peptide solution (5 pmol μL^−1^ parathyroid hormone peptide, and 25 pmol μL^−1^ muscarinic toxin 1 peptide) and 10 μL WCX binding solution were added to 5 μL serum. A 25 μL aliquot of this serum sample was mixed with 5 μL of WCX magnetic beads. The unbound fraction was separated from the beads using a magnetic bead separator and the beads were washed three times with 100 μL of wash buffer. Proteins and peptides were eluted from the magnetic beads using 10 μL each of an elution solution and a stabilization solution. Then, 1 μL of the peptide eluate was mixed with 10 μL of 2‐cyano‐4‐hydroxycinnamic acid matrix (Bruker Daltonics) and 0.8 μL of the mixture was spotted onto an AnchorChip target plate (Bruker Daltonics) and crystallized. Each sample was prepared in duplicate and four replicate spots were made from each elution, resulting in eight spots per sample. The eight spectra obtained were averaged for data analysis. Bead fractionation and sample spotting were performed automatically using the ClinProtRobot.

### 2.3 MALDI‐TOF MS

The AnchorChip target plate was placed in an Autoflex II TOF/TOF mass spectrometer (Bruker Daltonics) controlled by Flexcontrol 2.4 software (Bruker Daltonics). The instrument has a 337 nm nitrogen laser, delayed‐extraction electronics, and a 25 Hz digitizer and was externally calibrated using standard procedures. All acquisitions were obtained in LIFT mode using an automated acquisition method included in the instrument software and based on averaging 1000 randomized shots. The acquisition laser power was set between 20 and 35%. Spectra were acquired in positive linear mode in the mass range 600–10 000 Da. Peak clusters were completed using the second pass peak section (signal to noise ratio >5).

The relative peak intensities (normalized to a total ion current of between 1000 and 10 000 *m*/*z*) were expressed as arbitrary units. All measurements were performed using ClinProTools software 3.0 (Bruker Daltonics).

Five hundred laser shots from a total of 3000 laser shots were summed. The averaged MALDI‐MS/MS spectrum was subjected to a database search via the Mascot (Matrix Science, UK) database search engine using the search parameters: no enzyme specificity, 25 ppm mass tolerance for the parent mass, and 0.2 Da for the fragment masses. No fixed or variable modifications were selected. The NCBInr database was used for the search.

### 2.4 Data Analysis

#### 2.4.1 Conventional Method (Total Ion Current Method)

The relative peak intensities of *m*/*z* 1000–10 000 normalized to a total ion current were expressed as arbitrary units. All measurements were performed using ClinProTools software 3.0 (Bruker Daltonics GmbH).

#### 2.4.2 Assessment of the Ratio of the Peak Intensities Using Two Peptides as an Internal Standard for MALDI‐TOF MS

**Figure 1:**
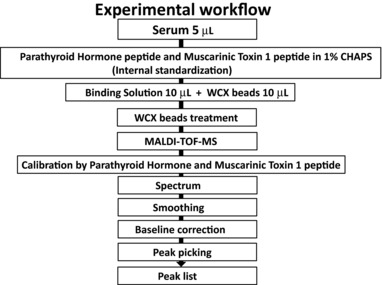
Experimental workflow for assessing the effect of preanalytical variables on serum peptidome profiling by MALDI‐TOF MS.

We used FlexAnalysis software 3.0 to perform baseline correction and smoothing. The peak intensities of serum spectra were obtained from the ratio of the peak intensity of serum to the peak intensity of two synthetic peptides. MS spectra ranging from *m*/*z* 600 to 10 000 obtained by quantifying peak intensities in the mass spectra of serum were obtained by mixing two synthetic peptides (parathyroid hormone and muscarinic toxin 1) into each sample. Assessment of the ratio of the peak intensity of MS spectra to the peak intensity of parathyroid hormone (MW, 3718) ranged from *m/z* 600 to 5999. The ratio of the peak intensity of MS spectra to the peak intensity of muscarinic toxin 1 peptides (MW, 7509) ranged from *m/z* 6000 to 10 000. We confirmed the number of MS peaks and peak conformance for 34 identified MS peaks, among all peaks at day 0, using peak picking. We made a list of the 34 MS peaks, among all peaks at day 0 (Figure 1). This process allowed us to evaluate the intensity of 34 MS peaks, among all observed peaks, following long‐term sample storage.

### 2.5 SDS‐PAGE Analysis

SDS‐PAGE analysis was conducted on 0.5 μL serum samples dissolved in PAGE sample buffer (50 mM tris‐HCL, pH 6.8 containing 50 mM dithiothreitol, 0.5% SDS, 10% glycerol) according to the equipment manufacturer's protocol (DRC Co., Ltd., Tokyo, Japan). The gel was stained with silver nitrate (Daiichi Pure Chemicals Co., Ltd., Tokyo, Japan) or Coomassie brilliant blue (CBB) (GE Healthcare, Little Chalfont, UK), then the gel image was converted to a densitogram using Scion Image.

### 2.6 Statistical Analysis

Relative peak intensity levels between groups were compared using the nonparametric Mann–Whitney U‐test and Kruskal–Wallis test with the Dwass–Steel–Critchlow–Fligner method. In all cases, *p* < 0.05 was considered statistically significant.

## 3 Results

### 3.1 Preparation of Internal Standards for MALDI‐TOF MS

**Figure 2:**
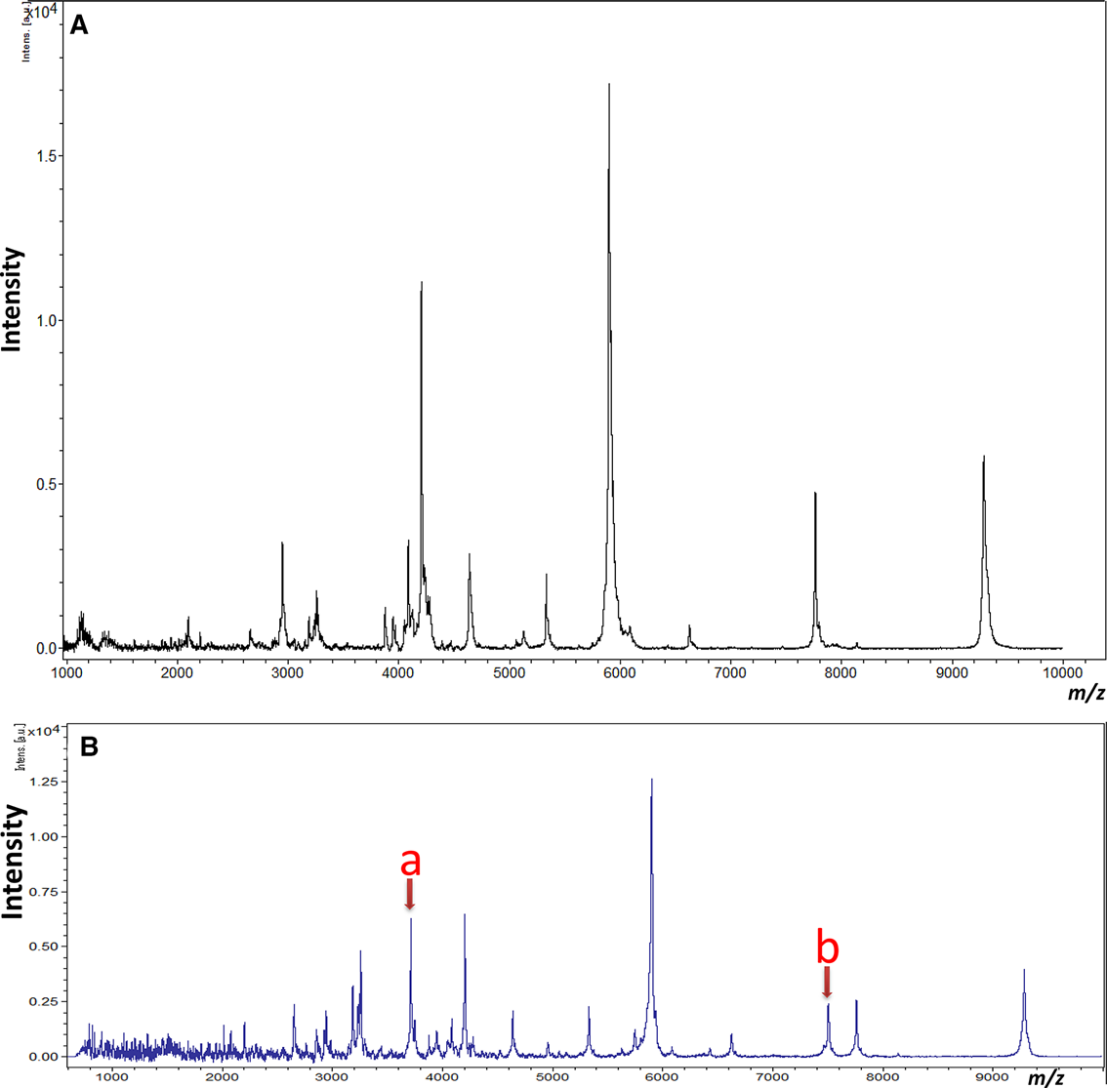
A) Representative spectrum of serum proteins and peptides obtained using ClinProt MALDI‐TOF MS. The relative peak intensities of *m*/*z* 1000–10 000 normalized to a total ion current were expressed as arbitrary units. All measurements were performed using ClinProTools software 3.0 (Bruker Daltonics). B) Representative spectrum of serum proteins and peptides obtained using ClinProt MALDI‐TOF MS spiked with (a) parathyroid hormone (MW, 3718) or (b) muscarinic toxin 1 (MW, 7509). Serum samples were collected from healthy volunteers.

The automated and robotic ClinProt system was used to evaluate the effects of long‐term storage parameters on the MS patterns of serum peptides (Figure 1). First, internal standards were prepared because peak intensities in mass spectra do not always reflect peptide concentrations in samples because of competition during the binding steps and variations in ionization efficiency. A stable isotope‐labeled internal standard dilution method is preferable to ensure accurate quantification of each peak in MALDI‐TOF MS^21^ and thus absolute quantification of the peak intensities in the mass spectra of serum were obtained by mixing two synthetic peptides (parathyroid hormone and muscarinic toxin 1) into each sample (Figure 2). The within‐run reproducibility of eight replicates was assessed. In preliminary experiments, the within‐run reproducibility coefficients of variation (CV) for the mass spectra of the parathyroid hormone and muscarinic toxin 1 peptides was low (2.5 and 2.7%, respectively), indicating that these synthetic peptides can be used as internal standards for accurate quantitation.

### 3.2 Evaluation of the Effects of Long‐Term Storage on the MS Patterns of Serum Peptides

The effects of serum storage temperature (−20 °C, −80 °C, or in liquid nitrogen) and storage duration (3, 6, or 12 months) on the MS patterns of serum peptides were evaluated. The number of peaks detected using the ClinProt system did not differ between samples stored at the three temperatures and for the different durations.

**Figure 3:**
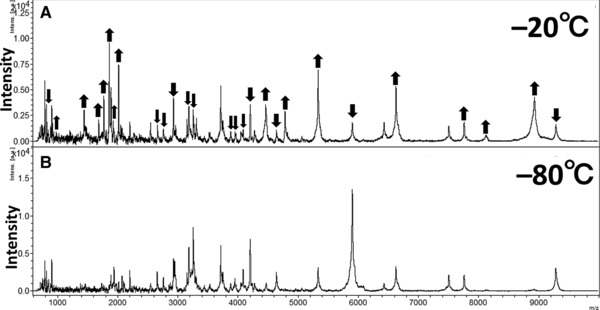
Comparison of MS patterns of serum peptide samples following long‐term storage (12 months) at −20 °C (A) and −80 °C (B). The MS patterns at day 0 were compared with those after each storage period. In panel (A) the up arrow (↑) represents significantly increased peak, and the down arrow (↓) represents significantly decreased peak.

**Figure 4:**
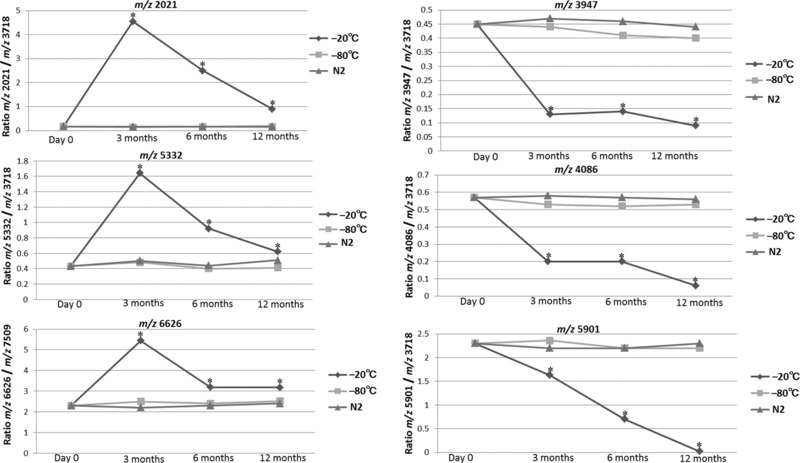
Comparative peptidome analyses of serum samples stored at −20 °C, −80 °C, or in liquid nitrogen, for 3, 6, or 12 months. MS patterns at day 0 were compared with those after each storage period. Presented ratios show the peak intensity of the serum sample, relative to that of the 3718 *m*/*z* parathyroid hormone, or that of the *m*/*z* 7509 muscarinic toxin 1 internal standards. Results are presented as a spaghetti plot., and show that freezing methods (−20 °C) dramatically affected MS peaks for storage times >3 months. A Kruskal–Wallis test, followed by Dwass–Steel–Critchlow–Fligner for multiple comparisons, was used to investigate statistical significance for peak intensities in the MALDI‐TOF MS analysis. We performed multiple testing adjustments. *p < 0.05 was considered statistically significant.

The MS patterns at day 0 were compared with those of the same donor serum sample after storage for 3, 6, and 12 months. After long‐term storage, 34 peaks with peculiar peak patterns were further evaluated (Figure 2, Figure 3 and Table 1). Following storage at −20 °C for 3 months, the intensities of the peaks at *m*/*z* 793, 905, 1686, 1774, 1861, 2021, 4462, 5332, 6626, 7761, and 9284 were significantly increased (Table 1 and Figure 4) whereas the intensities of the peaks at *m*/*z* 1938, 3877, 3947, 4086, 4205, and 5901 were significantly decreased (*p* < 0.05) (Table 1 and Figure 4).

**Table 1:** Comparative peptidome analyses of serum samples stored at −20 °C, −80 °C, or in liquid nitrogen for 3, 6, or 12 months

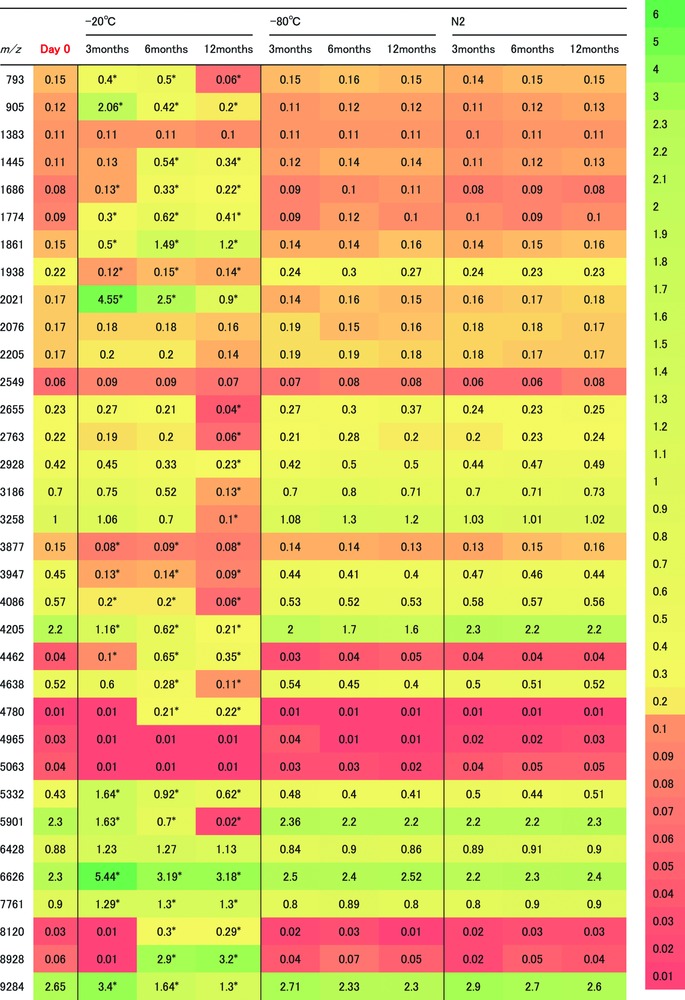

The MS patterns at day 0 were compared with those after each storage period. A heatmap of the ratios was produced to show the peak intensity of the serum sample to the peak intensity of the parathyroid hormone or muscarinic toxin 1 internal standard. Statistical significance of peak intensity in MALDI‐TOF MS analysis was determined using Kruskal–Wallis test with the Dwass–Steel–Critchlow–Fligner method. We performed multiple testing adjustment. *p* values of <0.05 were considered statistically significant. Color scale bar: maximum value = 6; minimum value = 0.01.

After storage at −20 °C for 6 months, the intensities of the peaks at *m*/*z* 1445, 4780, 8120, and 8928 were significantly increased (*p* < 0.05) (Table 1) while the intensities of the peak at *m*/*z* 4638 was significantly decreased (*p* < 0.05) (Table 1), and samples stored at −20 °C for 12 months provided peaks at *m*/*z* 2655, 2763, 2928, 3186, and 3258 whose intensities were significantly decreased (*p* < 0.05) (Table 1). In contrast, there were no differences in the detected MS patterns of samples stored at −80 °C or in liquid nitrogen for 3, 6, or 12 months (Table 1). These results indicate that the peak patterns remain unchanged for samples stored at −80 °C or in liquid nitrogen, whereas the intensities of as many as 20 MS peaks changed markedly during storage at −20 °C (Table 1).

We also assessed whether thawing conditions alter the relative intensities of serum peptide peaks. Samples stored at −80 °C or in liquid nitrogen for 3, 6, or 12 months were thawed using three conditions: kept on ice, at room temperature (around 25 °C), or in a 37 °C water bath. Thirty four MS peaks in day 0 aliquots were compared with aliquots of the same sample after 3, 6, and 12 months storage. There was no difference in the relative intensity of each peak between samples thawed on ice, at room temperature, or at 37 °C when stored for 3, 6, or 12 months at −80 °C or in liquid nitrogen (Supporting Information, Figure 1).

### 3.3 Comparison of Proteome Profiles Obtained by SDS‐PAGE and with Densitometry in Serum Samples Stored at Different Conditions

**Figure 5:**
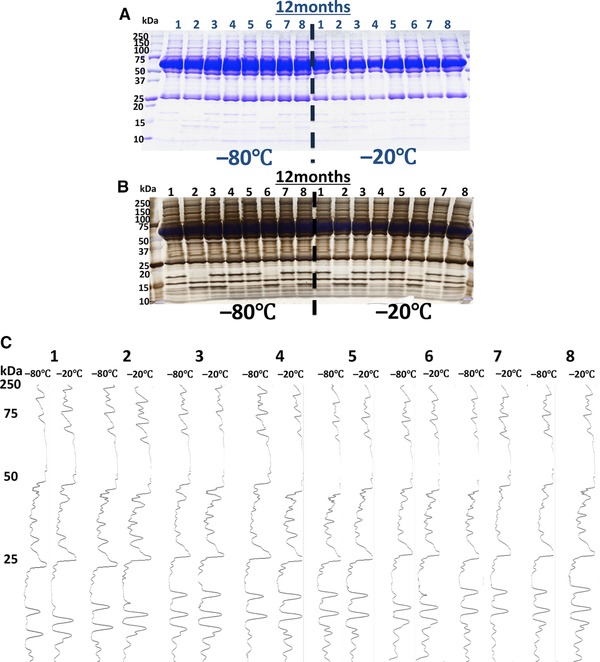
SDS‐PAGE with densitometric analysis of serum sample storage conditions. SDS‐PAGE patterns were visualized with (A) CBB or (B) silver staining. C) The results of densitometric analysis of the gels. Each peak corresponds to one protein band. Samples frozen at −20 °C and −80 °C were compared. The effects of temperature on long‐term storage (12 months) were also assessed.

Serum proteome analysis by SDS‐PAGE with densitometry was used to compare the effects of long‐term storage (12 months) at −20 °C and −80 °C; the results showed no significant differences in protein profiles as assessed by SDS‐PAGE (Figure 5A,B) or by densitometric analysis (Figure 5C; each peak corresponds to one protein SDS band).

## 4 Discussion

Serum peptide pattern analysis is a promising tool for identifying new disease‐associated biomarkers.^22^, ^23^ MALDI‐TOF MS can be used to generate profiles of low‐molecular‐weight peptides in clinical samples, including serum and CSF. Appropriate sample handling, however, is mandatory to obtain reproducible and proper results. Indeed, Del Boccio et al. reported a case in which a false conclusion was reached in a biomarker discovery investigation for multiple sclerosis because of inappropriate CSF storage conditions.^24^ Peak intensities in mass spectra, however, do not always reflect the true concentrations of proteins and peptides in samples due to variations in ionization efficiency. Important preanalytical variables include patient status such as fasted or postprandial, collection devices, processing time, storage temperature, and freeze–thaw cycles. Marko‐Varga et al. provided recommendations for standardizing the collection, processing, storage, and quality control of samples for long‐term storage, and Hsieh et al. reported that low‐molecular‐weight serum proteome components are affected by sampling, handling, and storage, with most changes resulting from inconsistent sampling procedures.^14^, ^25^

Several previous studies used MALDI‐TOF MS to assess the preanalytical stability of serum proteomes subjected to different storage conditions^12^, ^13^, ^14^ for a maximum period of 3 months. There is little clear information regarding how low‐molecular‐weight peptides in serum are affected by long‐term storage. Therefore, in the present study, we tested the effects of long‐term storage for up to 12 months on serum proteome and peptidome profiles. Furthermore, to date there have been no detailed investigations into how the proteome and peptidome patterns of serum samples stored at −20 °C differ from those stored at −80 °C. It is not uncommon for frozen serum samples to be stored at −20 °C instead of −80 °C in Japan and thus we focused on the effect of storage at −20 °C and −80 °C, as well as in liquid nitrogen, for a period of 12 months. Moreover, we quantitatively analyzed the peak intensities of peptides in the serum samples using two synthetic peptides (parathyroid hormone and muscarinic toxin 1) as internal standards for MALDI‐TOF MS analysis. The results of this study suggest that serum peptidome patterns obtained by MALDI‐TOF MS are affected by sample freezing and long‐term storage conditions.

In this study, internal standards were first prepared (Figures 1 and 2). The MALDI‐TOF MS system provided reproducible profiles of low‐molecular‐weight peptides in serum. The peak intensities in mass spectra do not always reflect the real amounts of serum peptides in samples due to the competition during binding steps and variations in ionization efficiency. The stable isotope‐labeled peptide standard dilution method is preferable for accurate quantitation of a particular MALDI‐TOF MS peak, as we previously reported.^21^ However, the major purpose of the present study was to assess changes in the number of peaks after long‐term storage. In the present study, we conducted serum peptidome profiling rather than absolute quantification of a single peak. Protein or peptide profiling by MALDI‐TOF MS is not necessarily reproducible^26^ and we need appropriate internal standards to evaluate the intensity of each MS peak. We believe that it is not possible to assess all peaks using only one specific stable‐isotope peptide as internal standard. Therefore, we tried to use some standard peptides as internal standards. Representative serum peptidome profile using a magnetic bead serum sample preparation for the ClinProt MALDI‐TOF MS system is shown in Figure 2A. It is obvious that very few MS peaks were seen at *m*/*z* between 3000 and 4000 and also between 7000 and 8000. We then obtained several peptides the molecular weights of which is either between 3000 and 4000 or between 7000 and 8000 as candidates for internal standard peptides.

Among these candidate peptides, the within‐run reproducibility (CV) for MS peak of the parathyroid hormone and muscarinic toxin 1 peptides, was 2.5 and 2.7%, respectively and were smaller than those of the four other peptides. Moreover, either double or triple MS peaks/peptides were observed in other peptides, whereas parathyroid hormone and muscarinic toxin showed sharp single MS peaks/peptides (Supporting Information, Figure 2). Based on these results, we decided to use parathyroid hormone and muscarinic toxin 1 peptides as internal standards. Parathyroid hormone was used as an internal standard for *m*/*z* 600–5999 and muscarinic toxin 1 was for *m*/*z* 6000–10 000.

Water freezes at 0 °C; however, in the presence of NaCl, the freezing point of water is lower at approximately −21 °C, known as the eutectic temperature.^27^, ^28^ This means that at −20 °C, water is not completely frozen if it contains NaCl. On the other hand, serum samples stored at −80 °C or in liquid nitrogen would have almost no liquid water; therefore, no hydrolysis would occur within 3 months.^29^ Consequently, no differences were detected in the MS patterns of samples stored at −80 °C or in liquid nitrogen for 3 months (Table 1). In contrast, the presence of a small amount of water could be sufficient to promote hydrolysis, even at −20 °C for 3 months. In the present study, the intensities of 11 peaks were significantly increased, and those of 6 peaks were significantly decreased (*p* < 0.05) (Table 1). Crystalized water enlarges and shrinks in response to changes in temperature. Ice crystals can affect the 3D structure of proteins, and such changes could have caused the denaturation observed in the serum samples stored for >3 months. Increased duration of storage at higher temperatures decreased the stability of the serum proteome. Hsieh et al. have shown that serum stored at 4 °C for 48 or 96 h exhibits significant changes in profile; however, serum stored at −80 °C for 3 months exhibits minimal changes in profile.^14^ Using SELDI‐TOF, serum storage at room temperature and at 4 °C for >2 months caused multiple peak differences.^14^ The effects of short‐term storage of serum and storage temperature were studied. Only minimal changes were observed in samples stored at room temperature within the first 4–6 h, whereas changes became observable after 8 h, particularly for peaks with *m*/*z* of approximately 3000. Hsieh et al. compared the proteomes of fresh serum samples with those stored at −80 °C for 1 and 3 months.^14^ They noted only minimal changes using magnetic bead‐based MALDI‐TOF MS.^14^ Rai et al. and Marshall et al. also reported similar results.^30^, ^31^ In this study, we also found that storage temperature had an important effect on serum protein profiles and that serum storage for 3 months at −80 °C preserved more serum proteins than storage at −20 °C. Interestingly, Willemse et al. investigated evaporation of various body fluids at different storage temperatures and storage durations.^32^ Serum samples were stored at different volumes (50, 250, 500, and 1000 μL) and at different temperatures (−80 °C, −20 °C, 4 °C, and room temperature) for 2 years.^32^ Serum storage at −80 °C or −20 °C protected serum samples from evaporation for >2 years.^32^ Therefore, it is unlikely that evaporation affected the serum samples used for MS profiling after storage at −20 °C for 3 months. However, it is important to note that serum contains a large number of soluble molecules, including many proteins that have varied stabilities.

Cryopreservation and freeze drying are the most common storage methods for serum samples. For cryopreserved samples, the materials are stored at approximately −20 °C or −80 °C in freezers or at −196 °C in liquid nitrogen, which is the ideal storage temperature. West‐Nielsen et al. reported that for short‐term storage, such as 7–14 d, it is advantageous to store samples at −80 °C rather than at −20 °C.^17^ Serum stored at 4 °C for 48 or 96 h showed significant profile changes, while serum stored at −80 °C for 3 months showed minimal profile changes.^14^ Samples stored at −20 °C might contain liquid water, which could promote hydrolysis. Seifarth et al. concluded that if long‐term storage is necessary, samples should be stored at −78 °C and must be thawed quickly.^33^ However, in our hands thawing conditions did not significantly influence serum pepitome patterns (Supporting Information, Figure 1). Samples stored at −80 °C have almost no liquid water, thereby preventing hydrolysis.^29^ In this study, serum stored at −20 °C for 3 months showed significant profile changes, while serum stored at −80 °C or in liquid nitrogen for 3 months showed minimal profile changes (Figure 5). These results indicate that the storage temperature has a significant influence on the quality of serum proteins and may be important for samples stored for long periods. The influence of storage temperature on the stability of serum samples was analyzed by MALDI‐TOF MS.

The results of this study showed significant differences in spectra at *m*/*z* 2022, 5901, 6626, 7760, and 9284 when stored at −20 °C for 3 months (Table 1). These spectra were submitted to a database search and the following peptides were identified: *m*/*z* 5901 (fibrinogen A*a* chain), 6626 (apolipoprotein C‐II), 7761 (apolipoprotein C‐I), 9284 (connective tissue‐activating peptide III [CTAP‐III]), and 2021 (complement C3f fragment) (Supporting Information, Table 1). Fibrinogen A*a* chain, apoC‐I, apoC‐II, CTAP‐III are reported to be involved in a variety of human diseases.

ApoC circulates in the blood as a component of chylomicrons, high‐density lipoproteins, and very‐low‐density lipoproteins,^34^, ^35^ and participates in the metabolism of lipoprotein particles.^34^, ^35^ Furthermore, apoC‐I is the activator of lecithin‐cholesterol acyltransferase, and overexpression of apoC‐I results in increased total cholesterol and triglyceride levels.^34^, ^35^ Takano et al. investigated novel factors involved in pancreatic cancer progression using a proteomics approach.^36^ Using surface‐enhanced laser desorption ionization/time‐of‐flight mass spectrometry, they compared pre‐ and postoperative serum protein profiles of pancreatic cancer patients who had curative pancreatectomy using and found that a high level of ApoC‐1 in preoperative serum significantly correlated with poor prognosis.^36^ The role of apoC‐II in lipoprotein metabolism and its potential effect on cardiovascular disease (CVD) have been investigated.^34^, ^35^ apoC‐II is an inhibitor of lipoprotein lipase and has been proposed as a risk factor for CVD.^37^

A 5.9 kDa peptide generated from the C‐terminal region of fibrinogen is used to detect gamma glutamyltransferase nonresponders in male subjects and may also be an early indicator of hepatic fibrosis in hepatitis C virus‐related chronic liver disease.^21^ CTAP‐III is a platelet‐associated chemokine that modulates tumor angiogenesis and inflammation and may be a potential biomarker of tumor growth.^38^, ^39^, ^40^ Using MALDI‐TOF MS, Yee et al. identified higher serum levels of CTAP‐III in pulmonary venous compared to arterial blood.^41^ In the present study, serum stored at −20 °C for 3 months showed significant changes in the intensities of these peaks, while serum stored at −80 °C or in liquid nitrogen did not (Table 1). The significant changes in the intensities of these peaks at −20 °C might be due to hyperactivation of the degradation process at −20 °C as compared to −80 °C or in liquid nitrogen.

Previous studies revealed that the method of sample preparation greatly influences MALDI‐TOF MS measurements.^30^, ^42^, ^43^ The most commonly used preparation method, the dried‐droplet method, involves depositing a mixture of sample matrix solution onto the surface of a target plate and evaporating the solvent. Previously, we reported that the dried‐droplet method at 40% relative humidity maximized the reproducible detection of peaks, suggesting that humidity control is essential for MALDI‐TOF MS sample preparation in clinical applications.^44^ In the present study, the temperature in the box was maintained at 25 °C and humidity was monitored using a HygroPalm thermohygrometer (Rotronic, Bassersdorf, Switzerland).

We observed no differences in protein profiles as assessed by SDS‐PAGE between samples stored at −20 °C or −80 °C (Figure 5A,B). High quality blood samples are needed for proteomic and peptidomic biomarker research, low quality samples may lead to false discovery, and thus the freezing and storage conditions may be very important. Our results suggest that further detailed observation of the effect of preanalytical variables, including blood sample storage conditions, is needed.

In summary, we used the automated robotic system (ClinProt system) to evaluate the effects of long‐term storage on the MS patterns of serum peptides. Storage conditions affected the serum peptidome MS profiles and the gel‐based protein profiles were unaffected by the freezing and storage temperature. The effect of preanalytical variables on the gel‐based and MS serum proteome and peptidome patterns suggest the importance of standardization for blood sampling and storage conditions for inter‐laboratory and multicenter studies using serum samples.

**Abbreviations**
MALDI‐TOF MS: matrix‐assisted laser desorption/ionization time‐of‐flight mass spectrometry
MS: mass spectrometry
SDS‐PAGE: sodium dodecyl sulfate polyacrylamide gel electrophoresis

## Conflict of Interest

The authors have declared no conflict of interest.

## Supporting information

Supporting Information
