# Supplementary material for: Assessment by Matrix‐Assisted Laser Desorption/Ionization Time‐of‐Flight Mass Spectrometry of the Effects of Preanalytical Variables on Serum Peptidome Profiles Following Long‐Term Sample Storage
Source: Proteomics Clin Appl. 2018 Mar 2;12(3):1700047. doi: 10.1002/prca.201700047 (PMC5947747; doi:10.1002/prca.201700047)
Supplement: Supplementary file 1 — Supporting Information [file PRCA-12-na-s001.pptx]

## Slide 1
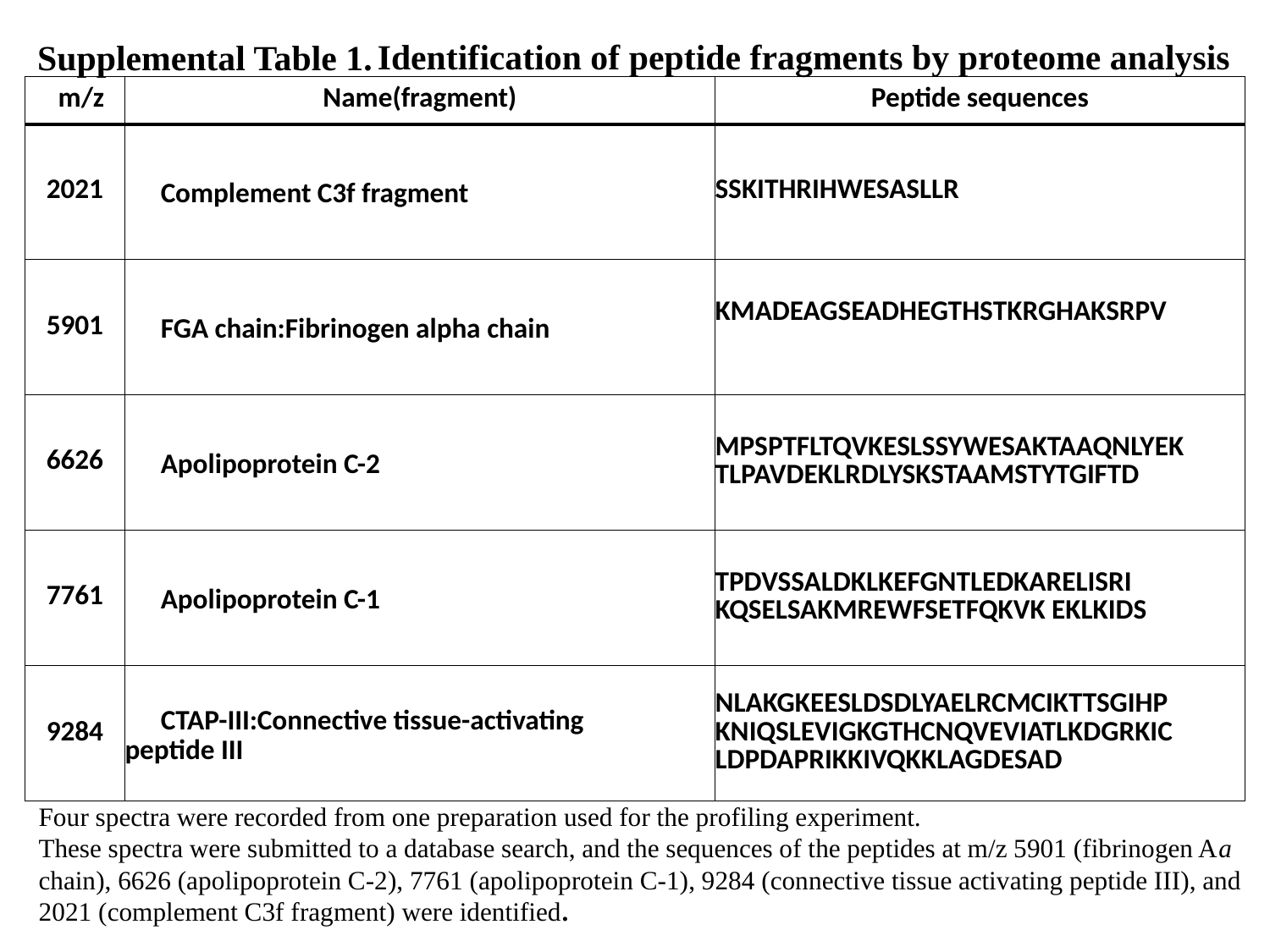

Supplemental Table 1.
Identification of peptide fragments by proteome analysis
| m/z | Name(fragment) | Peptide sequences |
| --- | --- | --- |
| 2021 | Complement C3f fragment | SSKITHRIHWESASLLR |
| 5901 | FGA chain:Fibrinogen alpha chain | KMADEAGSEADHEGTHSTKRGHAKSRPV |
| 6626 | Apolipoprotein C-2 | MPSPTFLTQVKESLSSYWESAKTAAQNLYEK TLPAVDEKLRDLYSKSTAAMSTYTGIFTD |
| 7761 | Apolipoprotein C-1 | TPDVSSALDKLKEFGNTLEDKARELISRI KQSELSAKMREWFSETFQKVK EKLKIDS |
| 9284 | CTAP-III:Connective tissue-activating　peptide III | NLAKGKEESLDSDLYAELRCMCIKTTSGIHP KNIQSLEVIGKGTHCNQVEVIATLKDGRKIC LDPDAPRIKKIVQKKLAGDESAD |
Four spectra were recorded from one preparation used for the profiling experiment.
These spectra were submitted to a database search, and the sequences of the peptides at m/z 5901 (fibrinogen Aa chain), 6626 (apolipoprotein C-2), 7761 (apolipoprotein C-1), 9284 (connective tissue activating peptide III), and 2021 (complement C3f fragment) were identified.

## Slide 2
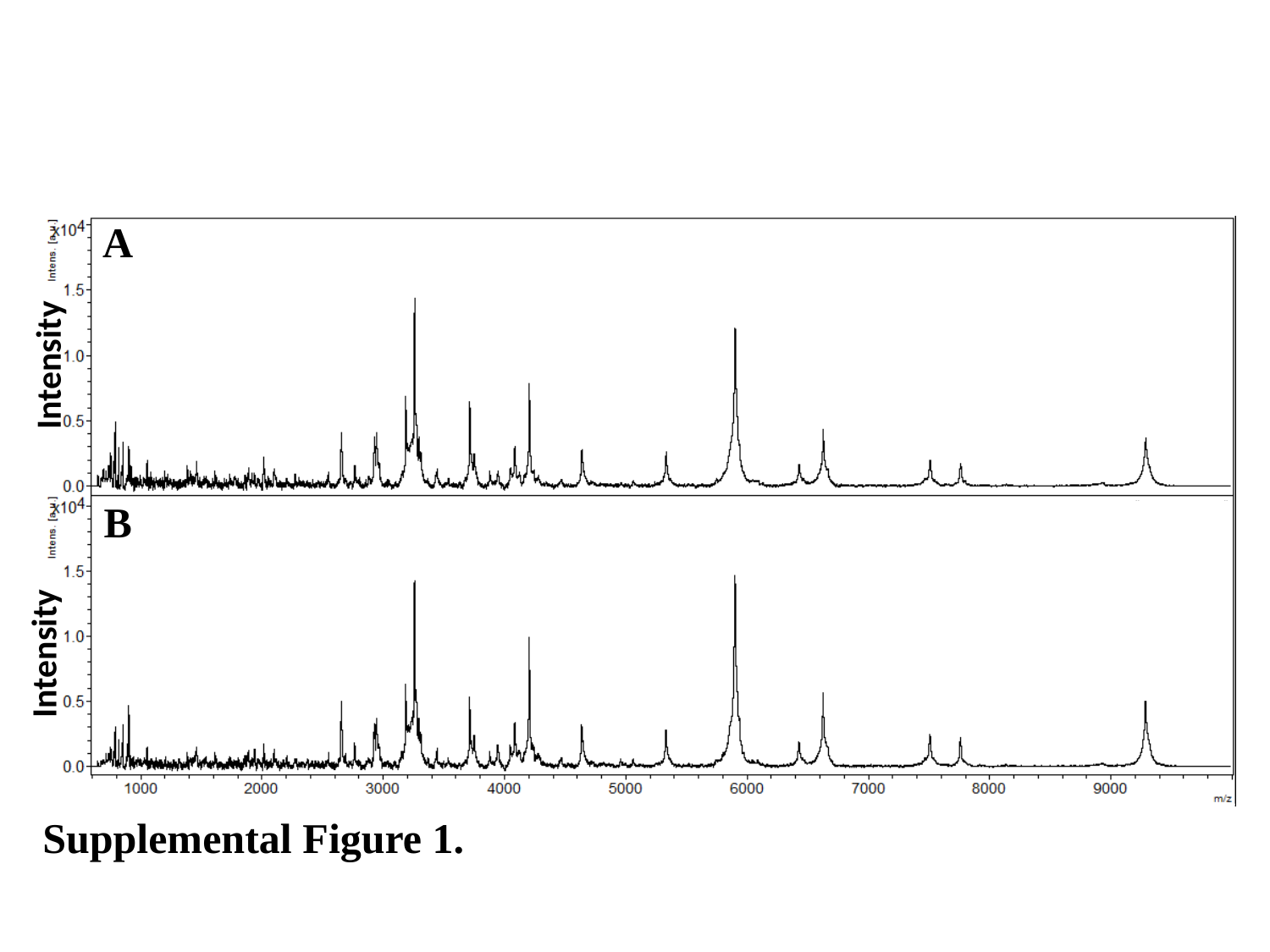

A
Intensity
Intensity
A
B
B
Supplemental Figure 1.

## Slide 3
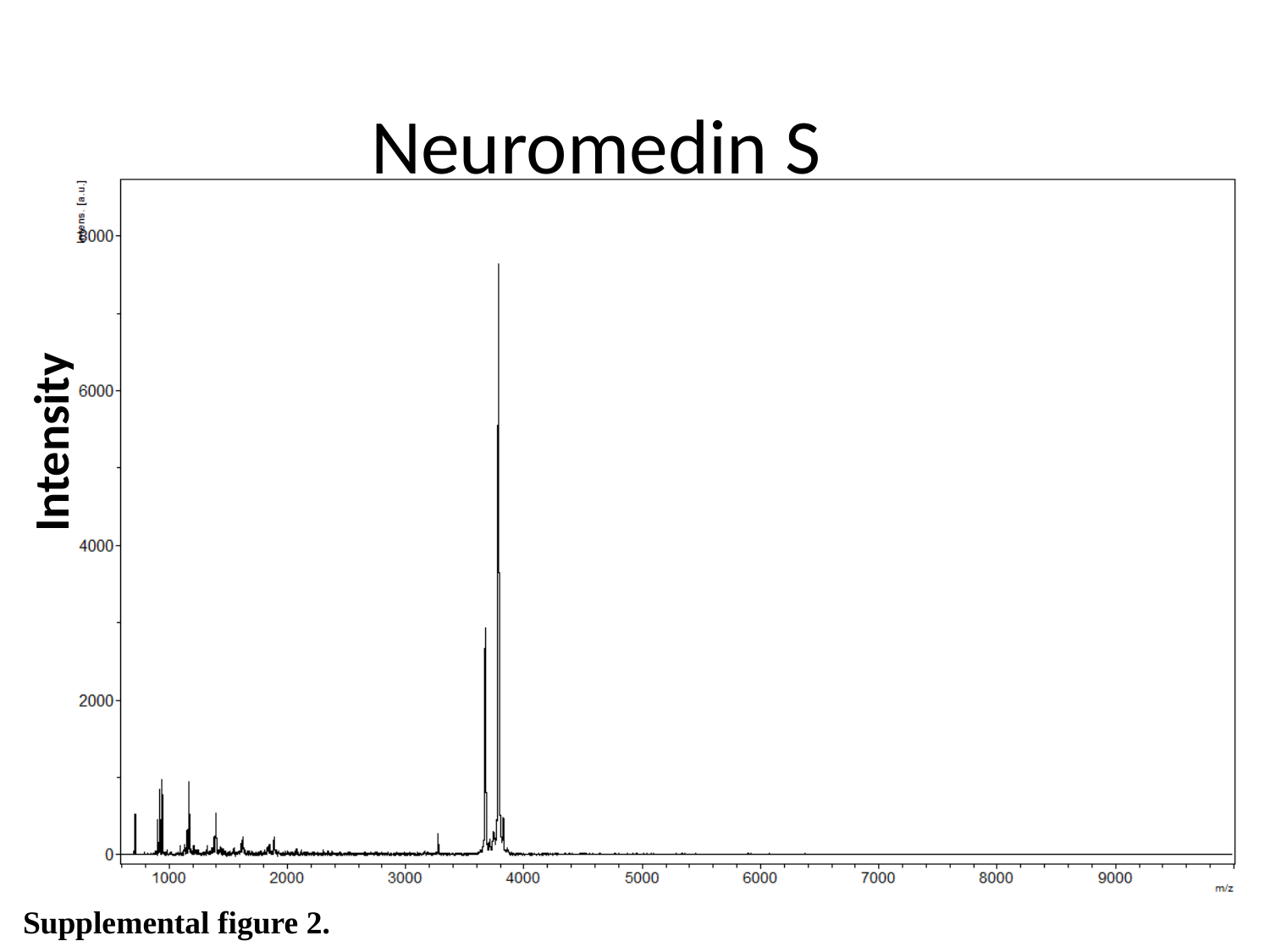

# Neuromedin S
Intensity
Supplemental figure 2.

## Slide 4
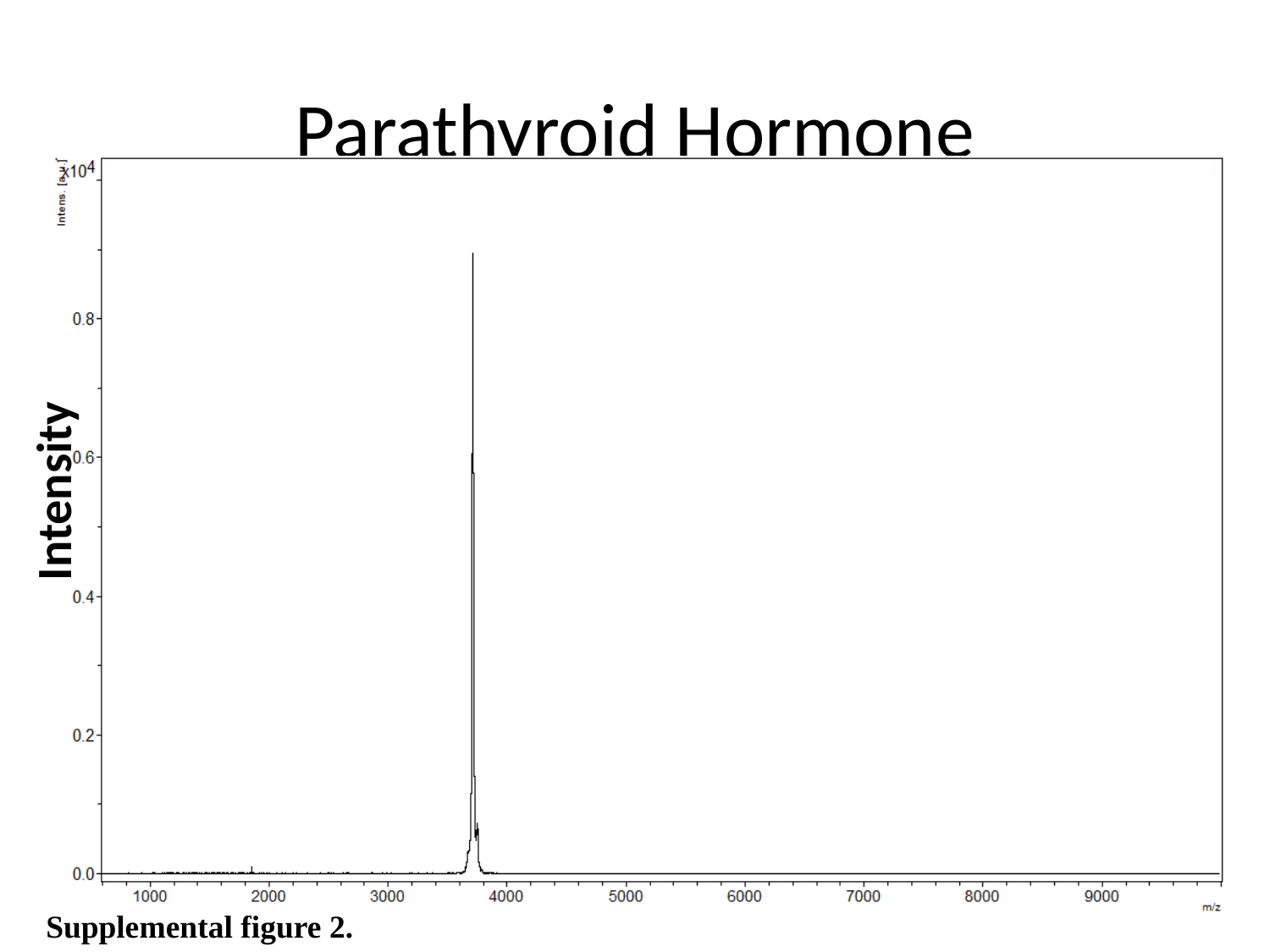

# Parathyroid Hormone
Intensity
Supplemental figure 2.
